# Supplementary material for: Dog Ecology and Rabies Knowledge of Owners and Non-Owners in Sanur, A Sub-District of the Indonesian Island Province of Bali
Source: Animals (Basel). 2018 Jul 5;8(7):112. doi: 10.3390/ani8070112 (PMC6070915; doi:10.3390/ani8070112)
Supplement: Supplementary file 1 [file animals-08-00112-s001.zip › animals-314792-Supplementary material 2 - KAP survey in English.pdf]

KAP SURVEY

Statement for consent, read out:

"As part of Program Dharma I would like to record some information about how you feel about dogs in your community. This information will be shared with Program Dharma team members to help us understand the wishes of your banjar. If other people outside the Program Dharma team would like to see this data we will ensure your name and any other identifiers are removed first, so that the data is anonymous. Are you happy for me to continue?"

<u>If they say no, thank them for their time and end the survey.</u>

INFORMATION ABOUT INTERVIEWEE

Your first and last name___

Name of banjar___

Name of person you are interviewing___

- Does this person own a dog?
- Are any of their dogs in your Dogalog?
- What is the name of their dog? (just one of their dogs in the Dogalog will do)

Date of survey___

GPS coordinates of household

LIKERT SCALES FOR ATTITUDE STATEMENTS

Please day how much you agree with the following statements, on a scale of strongly disagree, disagree, neutral, agree, strongly agree:

1. Mass culling of dogs is always necessary when there is one dog with rabies in my banjar
2. Imagine a dog that looks like it has symptoms of rabies is found in your banjar. Only the dog with rabies will be culled, all other dogs will be vaccinated.
3. I would prefer to vaccinate all the dogs in my banjar than cull them
4. It's important to me that dogs in my banjar are well cared for
5. Vaccinated dogs keep my community safe
6. Vaccinating dogs against rabies, protects people against rabies

DOG CARE SCENARIO

Imagine that a dog belonging to a family in your banjar becomes sick. It is thin, it has lost fur and its skin looks bad. What do you think should happen?

A. Dog should be killed
B. Dog should be taken outside the banjar
C. Nothing should be done, wait to see if it can survive
D. Dog should be left to see if it can survive on its own, but should not be kept with the family
E. The family is responsible for getting advice on how to care for the dog, to see if it can be made better

I know who to call when I need to have dogs vaccinated against rabies

- Yes / No

Are you interested in adopting a dog?

- Yes / No

DOG BITE KNOWLEDGE

What should you do if you are bitten by a dog that you don't know? *Don't prompt them. Tick any of the following that they mention*:

1. Wash the wound
2. Wash wound with running water
3. Wash wound with soap and running water
4. Wash wound for 15 minutes
5. Apply iodine or betadine
6. Notify T1 or T2
7. Notify Klian
8. Got to bite clinic or hospital
9. Get vaccinated
10. Act immediately
11. Don't get vaccinated if you are ill
12. Don't get vaccinated if you are pregnant
13. Do nothing unless dog tests positive
14. Wait for symptoms before acting
15. Use traditional medicine
16. Ask the Priest for help

DOG VACCINATION AND SYMPTOMS KNOWLEDGE

How often should dogs be vaccinated against rabies? *Don't prompt them, choose which ever fits their answer most closely*

A. Once in a lifetime
B. Every 3 years
C. Every year
D. Every month
E. Don't know
F. Never
G. Depends on the dog
H. Only if bitten by another dog

Can you list the symptoms of rabies in dogs?

1. Defensive aggressive
2. No appetite
3. Abnormal licking water
4. Drooping Jaw Tongue
5. Diarrhoea

6. Skinny Dog
7. Vomiting
8. Strange Movements
9. Biting eating abnormal object
10. Bad skin
11. Lethargic
12. Behavior change
13. Salivate
14. Abnormal bark
15. Running restlessness
16. Unprovoked bite
17. Avoids water
18. Avoids light
19. Avoids certain foods
