# Supplementary material for: Dog Ecology and Rabies Knowledge of Owners and Non-Owners in Sanur, A Sub-District of the Indonesian Island Province of Bali
Source: Animals (Basel). 2018 Jul 5;8(7):112. doi: 10.3390/ani8070112 (PMC6070915; doi:10.3390/ani8070112)
Supplement: Supplementary file 1 [file animals-08-00112-s001.zip › animals-314792-Supplementary material 1 - Dog demography questionnaire in English.pdf]

DOG DEMOGRAPHY QUESTIONNAIRE

Statement for consent, read out:

"As part of Program Dharma I would like to record some information about yourself and your dog(s). This information will be shared with Program Dharma team members to help us understand the needs of the dogs in your banjar. If other people outside the Program Dharma team would like to see this data we will ensure your name and any other identifiers are removed first, so that the data is anonymous. Are you happy for me to continue?"

If they say no, you can continue to talk to them about their dog but you must not enter their data into the app.

Address – enter banjar name and sector number

INFORMATION ABOUT THE DOG – FILL IN THE FIRST TIME THE DOG ENTERS THE DOGALOG

Date of entry

Dog's name (If owned, enter dog's name and owners surname; if unowned, enter stray and basic description, e.g. brown female)

Description of dog (free text)

Photo of dog

Gender of dog

- Male
- Female

Type of dog

- Breed dog
- Mixed breed
- Bali dog

Is this dog owned? Y/N

IF OWNED, ENTER INFORMATION ABOUT THE OWNER:

- First and last name of the owner
- Telephone number

Where did you get this dog from?

- Pup own dog
- Gift within desa
- Gift outside desa
- Purchase within desa
- Purchase outside desa
- Adopt street within desa

- Adopt street outside desa
- Adopt shelter
- Other

If gift, purchased or adopted outside desa, which desa did the dog come from?

- Denpasar
- Badung
- Bangli
- Buleleng
- Gianyar
- Jembrana
- Karangasem
- Klungkung
- Tabanan
- From outside Bali
- Don't know

How old is this dog on the day he recorded in Dogalog? Make it as precisely as possible. Leave blank if the age is not known. (Age is years + months, e.g. a 6 month old dog is 0 years and 6 months)

- Years
- Months

IF UNOWNED DOG

The estimated age of the dog on the day it enters the Dogalog

- Puppy < 4 months
- Juvenile 4-12 months
- Adult 1-7 years
- Old 7+ years

T1/2 NAME AND LOCATION

Full name of T1 or T2 filling in Dogalog (from drop down list, or free text if not on list)

Name of banjar (from drop down list)

Geolocation (automatic GPS location, provides lat long of current position)

REVIEW INFO AT EACH VISIT AND ONLY AMEND IF SOMETHING HAS CHANGED

Is this dog sterilised? If no one knows assume 'No' Y/N

Is the owner requesting sterilisation? If the dog is unowned enter 'Yes' Y/N

Is the dog vaccinated? If no one knows assume 'No' Y/N

What is the date of the most recent vaccination, leave blank if you do not know

(This date of most recent vaccination is followed by an automatic field that will display the days until revaccination is due, assuming 12 months vaccination and using today's date)

Additional comments (free text field)

HOW IS THIS DOG TODAY – OPENS THE DOGSTATUS FORM, ONE FORM FOR EACH VISIT

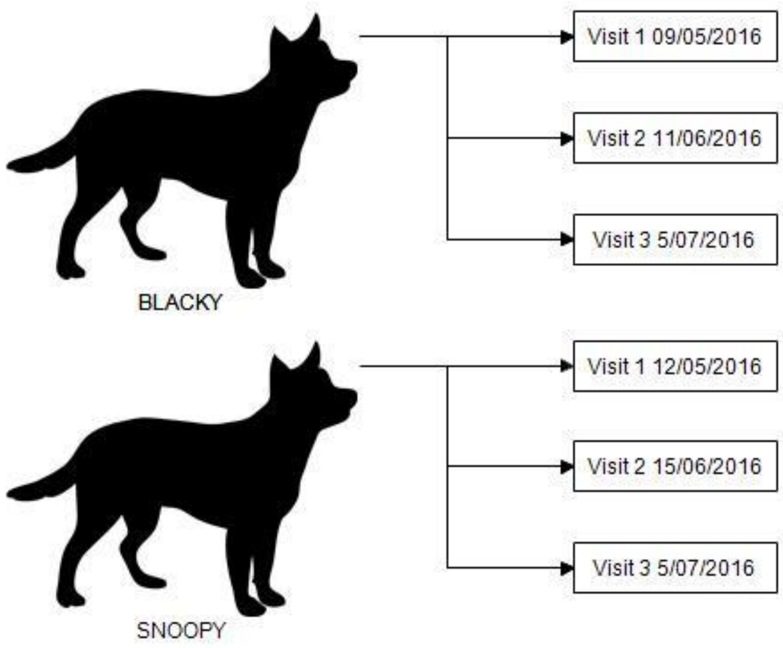

1 Dogalog per dog, several dogStatus for each dog, one for each visit

Date of visit (same as ‘Date of entry’ for first visit and Dogalog creation)

Tick if owned dog has left the household ☐
- If left, what was this dog’s fate?
  - Died
  - Euthanised
  - Killed by authorities
  - Killed by owner
  - Sold in banjar
  - Sold outside banjar
  - Sold meat trader
  - Gift in banjar
  - Gift outside banjar
  - Disappeared
  - Other

Tick if unowned dog has left the banjar ☐
- If left, what was this dog’s fate?
  - Died
  - Euthanised
  - Killed by authorities
  - Adopted within banjar

- Adopted outside of banjar
- Taken by a meat trader
- Disappeared
- Other

If dog has left, end DogStatus here, otherwise continue

Tick if not is not available to score for welfare and behaviour ☐

- If available, score for welfare:
  - Visible skin condition? Y/N
  - What is the dog's body condition?
    1. Emaciated
    2. Thin
    3. Ideal
    4. Fat
    5. Ideal
  - Are there any visible injuries? Y/N
  - How is the dog confined?
    1. Allowed to roam freely
    2. Confined in yard
    3. Confined in the house
    4. Confined in a kennel/cage
    5. Tethered
    6. Other
  - If caged or tethered…
    1. Does the dog have access to shade
    2. Does the dog have access to water
- If available, score for behaviour:
  - Ask the dog owner to call his dog over and pet them (touching a friendly way). If the dog does not come over, they can approach the dog to pet him. How does the dog react?
    A. Ignores
    B. Nervous
    C. Aggressive
    D. Positive
    E. The owner refuses to try
  - Once the owner has tried, or if this is an unowned dog, you (T1 or T2) do the same thing. Call the dog and try to pet them. If the dog does not come, you can approach the dog to pet him. Do not corner the dog. If the dog shows signs of aggressive, you must stop. How does the dog react to you?
    A. Ignores
    B. Nervous
    C. Aggressive
    D. Positive
    E. You refuse to try

IF THIS IS A FEMALE DOG, COMPLETE THEIR BREEDING STATUS/HISTORY:

- Tick if she is currently pregnant ☐
- Tick if she is currently lactating ☐

- Tick if she had puppies in the last 12 months ☐

DOES THIS DOG REQUIRE VETERINARY ASSISTANCE BEFORE THE NEXT REGULAR VISIT BY THE MOBILE CLINIC?

Tick if dog need assistance IMMEDIATELY ☐

Tick if dog needs assistance within the next week ☐

What is the situation that requires immediate assistance?
- Suspected rabies
- Dog bite that needs investigation – either the dog has bitten a person or another dog
- Poisoning
- Puppy: vomiting, diarrhoea or not eaten for more than one day
- Adult: vomiting, diarrhoea or not eaten for more than three days
- Blood in vomit of faeces
- Large infected wound
- Broken bone
- Bleeding heavily
- Collapsed, weak or shaking
- Seizures
- Trouble breathing
- Snake bite
- Other

What is the situation that requires assistance within a week:

- First vaccination
- Small wound
- Behaviour problem
- Distemper
- Apparently unowned lactating female roaming in the street
- Dumped litter of puppies
- Discharge from nose or eyes
- Coughing
- Puppy with rickets
- TVT or genital wound
- Serious skin problem
- Other

Tick if the owner thinks a new dog has arrived in the banjar since your last visit ☐

END
